# Post-infectious autoimmune disorder involving the CNS and PNS following SARS-CoV-2 infection – a clinical-morphological case report

**DOI:** 10.1186/s42466-025-00436-6

**Published:** 2025-10-15

**Authors:** Vincent Umathum, Carolin König, Dirk Bandorski, Lukas Scheffer, Joachim Weis, Heidrun H. Krämer, Jens Allendörfer, Anne Schänzer

**Affiliations:** 1 Institute of Neuropathology, Justus-Liebig-University Giessen, Giessen, Germany; 2 Institute of Pathology and Molecular Pathology, Bundeswehrkrankenhaus Ulm, Ulm, Germany; 3 Asklepios Neurological Clinic Bad Salzhausen, Nidda, Germany; 4 Institute of Pathology, Cytology, Dermatopathology and Molecular Pathology, Wetzlar, Germany; 5 Institute of Neuropathology, University Hospital RWTH Aachen, Aachen, Germany; 6 Department of Neurology, Justus-Liebig-University Giessen, Giessen, Germany; 7 Translational Neuroscience Network (TNNG), Justus-Liebig-University Giessen, Giessen, Germany

**Keywords:** Post-COVID, Neuritis, SARSCoV-2, COVID-19, Fatigue, Viral infection

## Abstract

Post-COVID can be associated with neurological symptoms such as neuropathy or fatigue. Histological analysis of affected nerves is rarely described and post mortem study of the brain and peripheral nerves are carried out only in few cases.

In the case report, we describe the clinical and neuropathological findings of a 75-year-old female patient who presented with a motor and sensory axonal neuropathy after a SARS-CoV-2 infection seven weeks before and suddenly died. Laboratory testing of serum and CSF revealed no signs of systemic vasculitis or infectious diseases. A post mortem investigation was performed. Samples from the brain including the cranial nerves, peripheral nerves and skeletal muscles from different regions were analysed.

The brain revealed lymphocytic cells predominantly in the basal ganglia and brain stem with involvement of the cranial nerves. Interestingly, a CNS involvement was not observed during lifetime. The sensory and motor peripheral nerves revealed a severe axonal neuritis. Skeletal muscle showed a neurogenic atrophy.

This case report highlights that a post-infectious autoimmune disorder with CNS and PNS involvement should be considered in patients with post-COVID. The diagnosis of an ongoing inflammation may influence the treatment options.

**Supplementary Information:**

The online version contains supplementary material available at 10.1186/s42466-025-00436-6.

## Case presentation

**Fig. 1:**
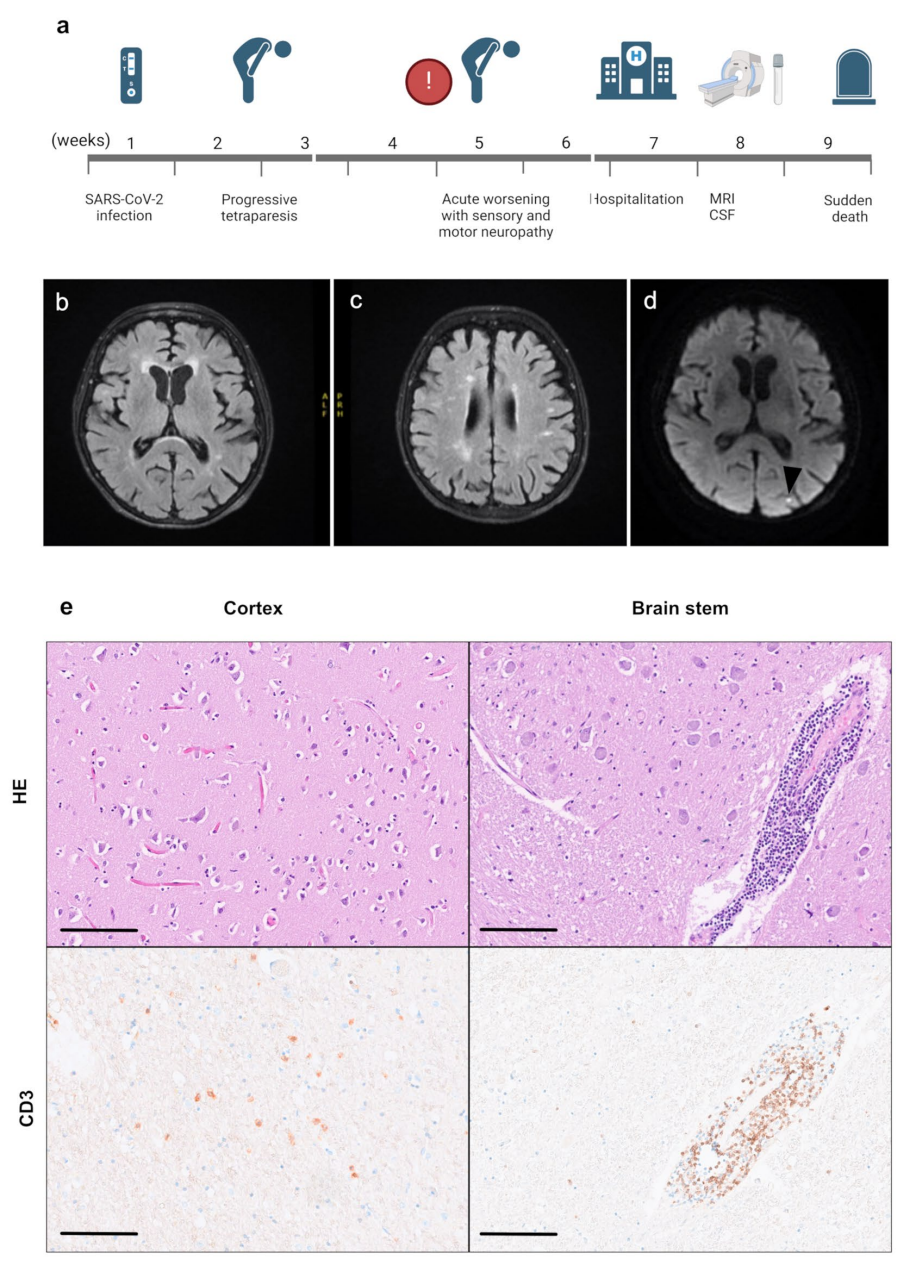
Time course, radiologic and histological findings in the brain: Time course of the clinical history (**a**). cMRI reveals WMH classified as Fazekas grade II, indicating the presence of cerebral small vessel disease. FLAIR sequences, highlighting WMH in the periventricular and deep white matter regions (**b**, **c**). DWI sequence with an acute, dot-like ischemic lesion located in the cuneus gyrus of the occipital lobe (indicated by the arrowhead) (**d**). CNS pathology shows T-lymphocytic (CD3) infiltrates with predominantly involvement of the brain stem (Scale bar = 50 μm) (**e**). cMRI = cranial Magnetic resonance imaging; CSF = Cerebrospinal fluid; WMH = white matter hyperintensities; FLAIR = Fluid Attenuated Inversion Recovery; DWI = Diffusion-weighted imaging; HE = Haematoxylin & Eosin. Figure (a) is created with biorendere

A 75-year-old woman reported holocranial headache, tinnitus, dizziness, progressive upper limb weakness and glove-shaped hypesthesia. The patient had been infected with SARS-CoV-2 with pulmonary symptoms seven weeks before (Fig. 1a).

The patient presented a symmetric proximal tetraparesis, absent Achilles reflexes, and reduced patellar and arm reflexes. Nerve conduction study indicated mild axonal neuropathy with few signs of demyelination. Muscle sonography revealed frequent fasciculations despite normal muscle echogenicity. Neurological examination revealed no abnormalities of the cranial nerves.

Cerebrospinal fluid (CSF) showed mild lymphocytic pleocytosis and elevated protein levels, as well as intrathecal IgG synthesis. Serum and CSF testing revealed no systemic vasculitis or infectious diseases. Ganglioside antibodies were negative. Creatine kinase level was normal. CMRI (native T1, T2, T2FLAIR, DWI and 3DTOF MIP without contrast agent) showed a small old ischemic lesion in the cuneus gyrus and white matter hyperintensities (Fazekas grade II) (Fig. 1b-d). Echocardiography identified a left atrial thrombus.

The neurological symptoms did not improve under methylprednisolone, which was administered intravenously at a dose of 500 mg over 3 days starting on the 8th day of hospitalization. Twelve days after admission, the patient was found asystolic. Immediate resuscitation was unsuccessful.

Post-mortem analysis revealed lung emphysema, chronic bronchitis, mild aspiration bronchial pneumonia, an acute myocardial infarction and non-stenosing coronary artery sclerosis.

**Fig. 2:**
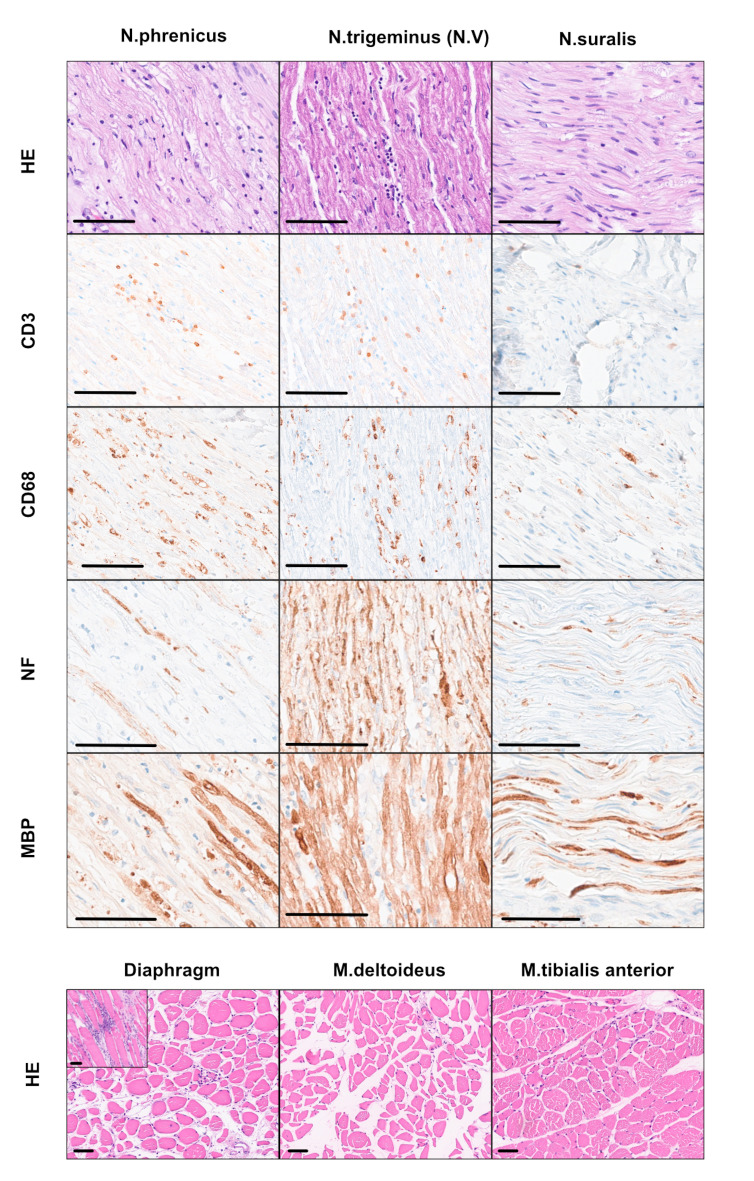
Inflammation in the nerves and skeletal muscles: Cranial and peripheral nerves show a strong T-cell (CD3) neuritis with macrophages (CD68) associated with axonal loss (NF) and loss of myelinated axons (MBP) consistent with a progressive inflammatory axonal neuropathy. Skeletal muscle involvement with inflammatory features in the diaphragm and neurogenic atrophy of deltoid and tibial muscle. HE = haematoxylin & eosin, NF = neurofilament, MBP = myelin basis protein (Scale Bar = 50 μm)

Neuropathological examination of the central nervous system (CNS) and peripheral nervous system (PNS) showed multifocal T-lymphocytic infiltrates predominantly perivascular in the brainstem and basal ganglia, associated with reactive astrogliosis and microglial activation (Fig. 1e). In addition, lymphocytic infiltrates were present in the cortical areas, but to a lesser extent than in the brainstem. An old lacunar ischemic lesion was seen in the corpus callosum. Severe inflammation associated with axonal loss and macrophagic invasion was present in the cranial (phrenic > trigeminal, vagus) and peripheral nerves (radial, sural, and sciatic) (additional file 1). Skeletal muscle morphology (quadriceps, deltoids, and tibialis anterior) was consistent with neurogenic atrophy. The diaphragm displayed a necrotizing inflammatory myopathy, likely related to phrenic neuritis (Fig. 2). SARS-CoV-2 spike protein could not be detected by immunohistochemistry in lung, CNS, or PNS tissue.

The likely cause of death in the patient was arrhythmogenic heart failure following acute myocardial infarction, potentially linked to severe axonal neuropathy of the vagus nerve. Additionally, severe axonal neuropathy and necrotizing myopathy of the diaphragm, may have worsened the symptoms.

## Discussion

This case report provides a post-COVID associated post-infectious autoimmune disorder involving the CNS and PNS. These symptoms are consistent with the theory that neurological symptoms associated with SARS-CoV-2 infection may be due to immune-mediated responses rather than direct viral invasion [1, 2]. About 40% of hospitalized COVID-19 patients report persisting neurological symptoms, including fatigue, paraesthesia, small fibre neuropathy, mononeuritis, demyelinating polyneuropathies like Guillain-Barré syndrome (GBS) and Miller Fisher syndrome (MFS), polyneuritis cranialis, and neuralgic amyotrophy [3]. It is not always possible to make a clear clinico-pathological distinction between the syndromes. Therefore, a broad diagnostic workup is required, particularly in cases involving a history of SARS-CoV-2 infection [4].

Neuropathological examination revealed inflammation predominantly in the brainstem and basal ganglia associated with neuritis of the trigeminal (N.V) and vagus (N.X) nerves. SARS-CoV-2 is thought to enter the brain via the olfactory mucosa or the vagus nerve, following specific neuroanatomical pathways, with viral presence most commonly detected in brainstem nuclei [2, 5].

Consistent with our findings, CNS pathology in COVID-19 patients includes interstitial brainstem inflammation (25%), accompanied by perivascular parenchymal T-cells (17%), and hypoxic-ischemic changes, including neuronal loss (25%) [6]. No other CNS pathology, such as micro-thrombosis or petechial haemorrhage, was present in our patient [7]. The majority of neuropathological studies are related to acute disease. However, persistent symptoms may impair everyday functioning even after a mild course of the disease, and functional and structural brain abnormalities have been linked to cognitive dysfunction [8]. Interestingly, in the patient, a CNS and cranial nerve involvement was not diagnosed ante mortem.

The patient presented with severe axonal neuritis affecting the motor and sensory nerves. Although SARS-CoV-2-associated neuropathies most often present as GBS-like demyelinating neuropathies [9]. Axonal neuropathies have only been described in few cases so far [10]. However, detailed morphological analysis of sensory and motoric nerves in post-COVID is rare.

In summary, we present the clinical and neuropathological findings of a patient with a SARS-CoV-2 associated post-infectious autoimmune disorder involving the CNS and PNS. These findings may enhance the understanding of post-COVID associated neurological symptoms and highlight the importance of morphological investigations to better classify the underlying course of the disease and improve treatment strategies.

## Supplementary Information

Below is the link to the electronic supplementary material.

Supplementary Material 1

## Data Availability

The data supporting the conclusions of this article are included within the article. Data not published within this article are available from the corresponding author on reasonable request.

## Abbreviations

cMRI: Cranial Magnetic Resonance Imaging
CNS: Central Nervous System
COVID-19: Coronavirus Disease 2019
CSF: Cerebrospinal Fluid
EMG: Electromyography
GBS: Guillain-Barré Syndrome
MFS: Miller Fisher Syndrome
PNS: Peripheral Nervous System
SARS: CoV-2-Severe Acute Respiratory Syndrome Coronavirus 2
