# Supplementary material for: Post-infectious autoimmune disorder involving the CNS and PNS following SARS-CoV-2 infection – a clinical-morphological case report
Source: Neurol Res Pract. 2025 Oct 15;7(1):76. doi: 10.1186/s42466-025-00436-6 (PMC12529787; doi:10.1186/s42466-025-00436-6)
Supplement: Supplementary file 1 — Supplementary Material 1 [file 42466_2025_436_MOESM1_ESM.docx]

|  | **T-Lymphoctye infiltration (CD3)** | **Macrophages**  **(CD68)** | **Axonal rarefication**  **(Neurofilament)** |
| --- | --- | --- | --- |
| **CNS** |  |  |  |
| Temporal Cortex | + | ++ |  |
| Basal Ganglia | ++ | ++ |  |
| Brain stem | ++ | ++ |  |
| **PNS** |  |  |  |
| N.I | (+) | + | ++ |
| N.II | + | ++ | + |
| N.V | + | ++ | ++ |
| N.VI, N.VII, N.VIII, N.IX | 0 | 0 | 0 |
| N.X | + | ++ | ++ |
| N.XI | + | + | ++ |
| N.phrenicus | ++ | ++ | ++ |
| N.radialis | + | ++ | + |
| N.suralis | + | ++ | + |
| N.ischiadicus | + | ++ | + |

**Additional file 1: Table 1:** Distribution of inflammation in the CNS, cranial nerves and PNS
